# Role of Cav-1 in HIV-1 Tat-Induced Dysfunction of Tight Junctions and A*β*-Transferring Proteins

**DOI:** 10.1155/2019/3403206

**Published:** 2019-05-14

**Authors:** Min Zou, Wen Huang, Wenlin Jiang, Yu Wu, Qiangtang Chen

**Affiliations:** ^1^Department of Neurology, First Affiliated Hospital, Guangxi Medical University, Nanning 530021, China; ^2^Guilin People's Hospital, Guilin 541000, China

## Abstract

**Objective:**

To evaluate the role of caveolin-1 (Cav-1) in HIV-1 Tat-induced dysfunction of tight junction and amyloid *β*-peptide- (A*β*-) transferring proteins.

**Methods:**

A Cav-1 shRNA interference target sequence was cloned into the lentiviral vector pHBLV-U6-Scramble-ZsGreen-Puro and verified by double enzyme digestion and DNA sequencing. Human cerebral microvascular endothelium (HBEC-5i) cells were transduced with viral particles made in 293T cells by transfection with lentiviral packaging plasmids. HBEC-5i cells transduced with Cav-1 shRNA or Ctr shRNA were exposed to HIV-1 Tat for 24 h, and the protein and mRNA levels of the tight junction protein occludin, A*β*-transferring protein, receptor for advanced glycation end products (RAGE), low-density lipoprotein receptor-related protein- (LRP-) 1, and RhoA were evaluated with Western blot and real-time reverse transcription polymerase chain reaction (qRT-PCR) assays, respectively.

**Results:**

After sequencing, an RNA interference recombinant lentivirus expressing a vector targeting Cav-1 was successfully established. The recombined lentiviral particles were made by using 293T cells to package the recombined lentiviral vector. A stable monoclonal cell line with strong GFP expression was acquired with a Cav-1 knockdown rate of 85.7%. The occludin protein and mRNA levels in the Ctr shRNA group were decreased with HIV-1 Tat exposure but were upregulated in the Cav-1 shRNA group. The HIV-1 Tat-induced alterations of RAGE and LRP-1 protein and mRNA levels in the Ctr shRNA group were attenuated in the Cav-1 shRNA group. The RhoA protein levels in the Ctr shRNA group were upregulated by HIV-1 Tat exposure but were downregulated in the Cav-1 shRNA group.

**Conclusion:**

These results show that HIV-1 Tat-induced downregulation of occludin and LRP-1 and upregulation of RAGE and RhoA may result in the accumulation of A*β* in the brain. Silencing the Cav-1 gene with shRNA plays a key role in the protection against HIV-1 Tat-induced dysfunction of the blood-brain barrier and A*β* accumulation.

## 1. Introduction

The cognitive neurological disorders caused by human immunodeficiency virus (HIV) are classified as HIV-associated neurocognitive disorders (HAND) [1]. After the introduction of highly active antiretroviral therapy (HAART), the incidence of severe cognitive impairment has dramatically decreased [2]. However, HAND persists in the era of potent antiretroviral therapy, and about half of all HAART-treated patients develop a mild cognitive impairment or asymptomatic neurocognitive impairment [3]. The mechanisms by which HIV infection provokes progressive neurocognitive impairment remain obscure. HAND is similar to those of Alzheimer's disease in that factors such as the amyloid/Tau pathways and stress-related pathways as well as blood-brain barrier (BBB) integrity [4]. Tight junctions (TJ) are comprised of different proteins such as occludin, claudin family members, zonula occludens (ZO) family members, and junctional adhesion molecules [5], which form a charged selective pore that only allows small ions and uncharged molecules to pass through. Occludin is located in the cytoplasmic membrane of endothelial cells in the brain and strongly influences vascular barrier function [6, 7]. Tight junction proteins are essential components of BBB integrity [8]. BBB dysfunction appears to be a particularly important component of HAND [9, 10]. Amyloid *β*-peptide (A*β*) accumulation, aggregation, and deposition in the brain are thought to be a causative pathological event in Alzheimer's disease as well as in HAND [11, 12]. A*β* biogenesis and clearance are potentially influenced by HIV viral infection [12]. Increased expression of amyloid precursor protein in neurons in AIDS has been documented [13]. Enhanced beta-amyloidosis and suppression of A*β* clearance by HIV infection of human primary macrophages have been found in ART-treated HAND and HIV-associated encephalitis brains [14]. HIV-1 may contribute to amyloid *β*-peptide accumulation in brain endothelial cells [15]. Impaired efflux and influx of A*β* across the BBB have been implicated in HIV viral infection. Our previous data show that HIV-1 Tat regulates the expression of occludin, the A*β* transfer receptors, receptor for advanced glycation end products (RAGE), and low-density lipoprotein receptor-related protein-1 (LRP-1) in brain endothelial cells via the Rho/ROCK signaling pathway [16]. Treatment with hydroxyfasudil, a specific inhibitor of ROCK, reverses the effect of decreased mRNA and protein levels of occludin and LRP-1 and reverses upregulation of RAGE mediated by the HIV-1 Tat protein [16].

Caveolae-associated signaling plays an important role in the disruption of tight junctions upon Tat exposure. Caveolins are the main structural proteins residing in the caveolae. The caveolae structure is composed of caveolin-1 (Cav-1), Cav-2, Cav-3, and four additional proteins known as Cavin 1-4. Cav-1 is the main marker of caveolae in endothelial cells. Evidence indicates that Cav-1 interacts with many signaling molecules, such as mitogen-activated protein kinases (MAPK) [17]. The expression of Cav-1 is upregulated by HIV infection in macrophages [18]. Exposure to HIV-1 Tat also results in the elevation of Cav-1 levels and activation of the Ras pathway; however, Cav-1 silencing markedly attenuates Tat-induced Ras expression in human brain microvascular endothelial cells [19].

However, the mechanisms of the interaction of HIV-1 Tat with caveolar membranes, BBB integrity, and A*β* accumulation are not fully understood. Therefore, the aim of the present study was to evaluate the role of Cav-1 in Tat-induced dysfunction of occludin and the A*β*-transferring proteins RAGE and LRP-1 in brain endothelial cells. Our novel observations indicate that HIV-1 Tat-induced downregulation of occludin and LRP-1 and upregulation of RAGE may result in the accumulation of A*β* in the brain. In addition, silencing the Cav-1 gene with shRNA protected against HIV-1 Tat-induced dysfunction of the blood-brain barrier and A*β* accumulation in brain endothelial cells.

## 2. Materials and Methods

### 2.1. Cell Cultures

Human cerebral microvascular endothelial cells (HBEC-5i) were purchased from American Type Culture Collection (ATCC, Manassas, VA, USA). The cells were cultured on 0.1% gelatin solution-coated (ATCC) flasks in Dulbecco's modified Eagle's medium (DMEM): F12 medium (ATCC) supplemented with 10% fetal bovine serum (FBS, Gibco/Thermo Fisher, Waltham, MA, USA), 40 *μ*g/mL endothelial cell growth supplement (ECGS, ATCC), and 100 U/mL penicillin as well as 0.1 mg/mL streptomycin (Beyotime, Shanghai), maintained at 37°C in a humidified atmosphere with 5% CO_2_ as described previously [20].

Human embryonic kidney (HEK) 293T cells (ATCC, Manassas, VA, USA) were grown in DMEM containing 10% FBS, 100 U/mL penicillin, and 0.1 mg/mL streptomycin (Beyotime, Shanghai), maintained at 37°C in a humidified atmosphere with 5% CO_2_.

### 2.2. Cav-1 Silencing and shRNA Lentiviral Particle Transduction

Cav-1 silencing was performed as described by Andras et al. [21] using Oligos targeting the human Cav-1 mRNA sequence 5′-CATCTACAAGCCCAACAACAGTGTA-3. Lentiviral particles contain target-specific constructs that encode shRNAs designed to knockdown Cav-1 expression. Cav-1 shRNA (5′-GatccGACGAGCTGAGCGAGAAGCAAGTGTATTCAAGAGATACACTTGCTTCTCGCTCAGCTCGTTTTTTTc-3′; 5′-aattgAAAAAAACGAGCTGAGCGAGAAGCAAGTGTATCTCTTGAATACACTTGCTTCTCGCTCAGCTCGTCg -3′) and negative control shRNA were purchased from Hanbio Biotechnology (Shanghai, China). In brief, the Cav-1 shRNA interference target sequence was cloned into the lentiviral vector pHBLV-U6-Scramble-ZsGreen-Puro and verified by double enzyme digestion and DNA sequencing. When HEK 293T cells reached 70~80% confluence, they were transfected with the lentiviral packaging plasmids (pSPAX2, pMD2G, and Cav-1 shRNA) using the LipoFiter™ Liposomal Transfection Reagent (Hanbio Biotechnology, Shanghai, China), and the viral particles (Cav-1 shRNA) were collected. The virus titers of lentivector were determined by green fluorescence under a fluorescence microscope using the whole dilution method. Transducing units (TU) per mL was calculated using the following equation: ((Percent positive GFP × number of cells)/100 × volume of viral dilution added to each well) × 1/dilution factor. HBEC-5i cells were transduced with the viral particles. Stable monoclonal cell lines with strong GFP expression were acquired and measured by fluorescence quantitative PCR (Q-PCR).

### 2.3. Cell Viability Assay

Puromycin was used for selection of the cells transduced with the Cav-1 shRNA lentiviral vectors because of the lentiviral vector pHBLV-U6-Scramble-ZsGreen-Puro having Pac gene. Recombinant HIV-1 Tat clade-B protein (amino acids 1 to 86) was purchased from ProSpec (Rehovot, Israel). The effects of puromycin and HIV-1 Tat on the viability of HBEC-5i cells were assessed by a Cell Counting Kit-8 assay (Biotech, Beijing, China). Briefly, a density of 1 × 10^4^ cells/well HBEC-5i cells was seeded onto 96-well plates. After 12 h, HBEC-5i cells were treated with puromycin at 0, 0.5, 1, 1.5, 2, 2.5, or 3 *μ*g/mL or Tat at 0, 0.5, 1.0, 1.5, 2, 2.5, or 3 *μ*g/mL for 24 h. Ten microliters of the CCK8 solution was added to each well of the plate and incubated at 37°C for 1 h. The absorbance at 450 nm was recorded using an iMark microplate reader (Bio-Rad, Hercules, CA) at 24 h.

### 2.4. Western Blot Analysis

Following treatment, cells were washed three times and lysed using the radioimmunoprecipitation assay cell lysis buffer (Beyotime) containing protease inhibitor cocktail tablets (Beyotime). Lysates were collected and centrifuged at 12000 g for 15 min, and protein levels were quantified using a BCA Protein Assay Kit (Beyotime). Next, 30 *μ*g protein was separated by SDS-PAGE and transferred onto polyvinylidene fluoride membranes (0.45 *μ*m; Millipore, Billerica, MA, USA). The membranes were blocked for 1 h with 5% fat-free milk at room temperature and then incubated with primary antibodies against occludin (1 : 1000, mouse monoclonal, 33-1500 in Thermo Fisher, Carlsbad, CA, USA), RAGE (1 : 1000, rabbit monoclonal; ab3611 in Abcam, Cambridge, MA, USA), LRP-1 (1 : 10000, rabbit monoclonal; ab92544 in Abcam), RhoA (1 : 1000, mouse monoclonal, ab54835 in Abcam), or GAPDH (1 : 5000, 10494-1-AP in Proteintech Group, Chicago, IL, USA) at 4°C overnight. The next day, the membranes were washed and incubated with IRDye 680 RD immunoglobulin (Ig) G (1 : 10000; goat anti-rabbit 926-68071 or goat anti-mouse 926-68070 in LI-COR Biosciences, Lincoln, NE, USA) secondary antibody for 1 h at room temperature. The detected proteins were then visualized using an Odyssey Infrared Imaging System (LI-COR Biosciences). Band density was analyzed by ImageJ software (National Institutes of Health, Bethesda, MD, USA), and signal density was calculated as the ratio of signal intensity to that of GAPDH.

### 2.5. Real-Time Reverse Transcription-Polymerase Chain Reaction

After treatment, the cells were harvested, total RNA was extracted using the TRIzol reagent (Takara Bio, Dalian, Japan), and cDNA was generated from 1 *μ*g RNA using the PrimeScript RT reagent kit (Takara) according to the manufacturer's instructions. Genomic DNA was removed with gDNA Eraser which was included in the RT reagent kit prior to reverse transcription reactions. cDNA was used for quantitative RT-PCR using a Taq PCR Master Mix kit (Takara) and conducted on the StepOnePlus Real-Time PCR System (Applied Biosystems, Foster City, CA, USA) using an RT Reaction Mix in a total volume of 20 *μ*L at 95°C for 30 s followed by 95°C for 5 s and 60°C for 30 s for 40 cycles. The primer sequences of occludin, RAGE, LRP-1, and GAPDH were as described previously [16]. GAPDH was used to normalize target gene mRNA levels, which were analyzed using the 2^–ΔΔCt^ method.

### 2.6. Statistical Analysis

Data were analyzed using SPSS 17.0 (SPSS, Chicago, IL, USA). The data are shown as means ± standard deviation. Comparisons between groups were conducted using one-way ANOVA, and Student-Newman-Keuls (SNK) was used for the post hoc test. Differences were considered statistically significant at *P* < 0.05.

## 3. Results

### 3.1. The Inhibition of Cav-1 by Cav-1 shRNA in HBEC-5i Cells

**Figure 1:**
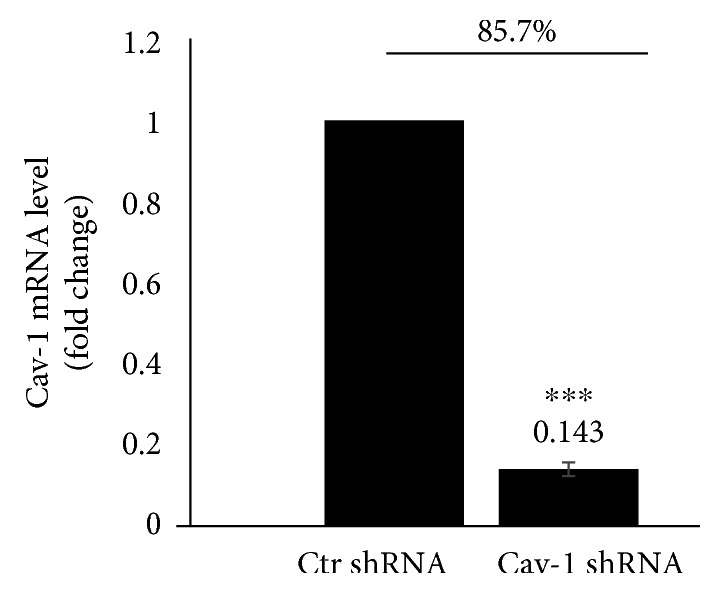
Cav-1 inhibition with shRNA. 293T cells were transfected with the lentiviral packaging plasmids (pSPAX2, pMD2G, and Cav-1 shRNA), and the viral particles (Cav-1 shRNA) were collected. The virus titer of lentivector was 2 × 10^8^ TU/mL, as assessed by green fluorescence under a fluorescence microscope using the whole dilution method. Cav-1 expression in HBEC-5i was silenced by transduction with Cav-1 shRNA and Ctr shRNA lentiviral vectors. The Cav-1 inhibition rate of the stable monoclonal cell lines was 85.7%, as detected by qRT-PCR. Data are representative of three independent experiments. ^∗∗∗^*P* < 0.001 versus control.

293T cells were transfected with the lentiviral packaging plasmids, and the viral particles were collected. The virus titer of lentivector was 2 × 10^8^ TU/mL, as assessed by green fluorescence under a fluorescence microscope using the whole dilution method. To examine the effect of Cav-1 on HIV-1 Tat-induced changes of tight junctions in the endothelium, HBEC-5i cells were transduced with lentiviral vectors of Cav-1 shRNA or negative control (Ctr shRNA). The Cav-1 inhibition rate of the stable monoclonal cell lines was 85.7%, as detected by qRT-PCR (Figure 1).

### 3.2. Cell Viability Assay

**Figure 2:**
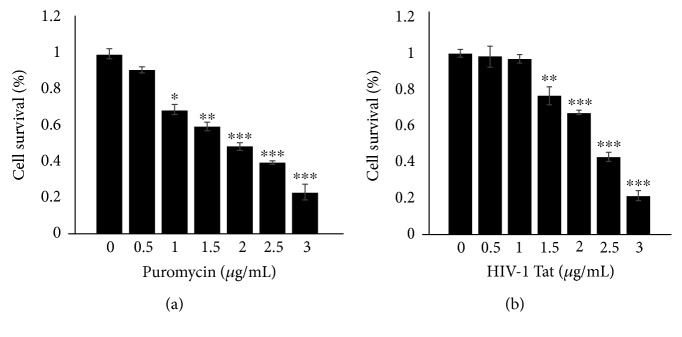
Cell viability assay. Effects of puromycin at 0, 0.5, 1.0, 1.5, 2.0, 2.5, or 3.0 *μ*g/mL for 24 h on the viability of HBEC-5i cells were assessed by a CCK8 assay. Cell viability was affected over 1 *μ*g/mL of puromycin (a). Cav-1 expression in HBEC-5i cells was silenced by transduction with lentiviral vectors of Cav-1 shRNA. Effects of HIV-1 Tat at 0, 0.5, 1.0, 1.5, 2.0, 2.5, or 3.0 *μ*g/mL for 24 h on the viability of HBEC-5i cells silenced with Cav-1 shRNA were also assessed by a CCK8 assay. Cell viability was not affected by 1 *μ*g/mL HIV-1 Tat for 24 h (b). Data are representative of three independent experiments. ^∗^*P* < 0.05, ^∗∗^*P* < 0.01, and ^∗∗∗^*P* < 0.001 versus control.

Puromycin was used for selection of the cells transfected with the Cav-1 shRNA lentiviral vectors, and HIV-1 Tat was applied to the following experiments. The effects of puromycin or HIV-1 Tat at 0, 0.5, 1, 1.5, 2, 2.5, or 3 *μ*g/mL for 24 h on the viability of HBEC-5i cells were assessed by a CCK8 assay. HBEC-5i cell viability was affected at over 1 *μ*g/mL of puromycin (Figure 2(a)). HBEC-5i cells were silenced by transfection with lentiviral vectors of Cav-1 shRNA. Cav-1 shRNA cell viability was not affected under 1 *μ*g/mL HIV-1 Tat for 24 h (Figure 2(b)).

### 3.3. The Role of Cav-1 shRNA in HIV-1 Tat-Induced Changes of Occludin

**Figure 3:**
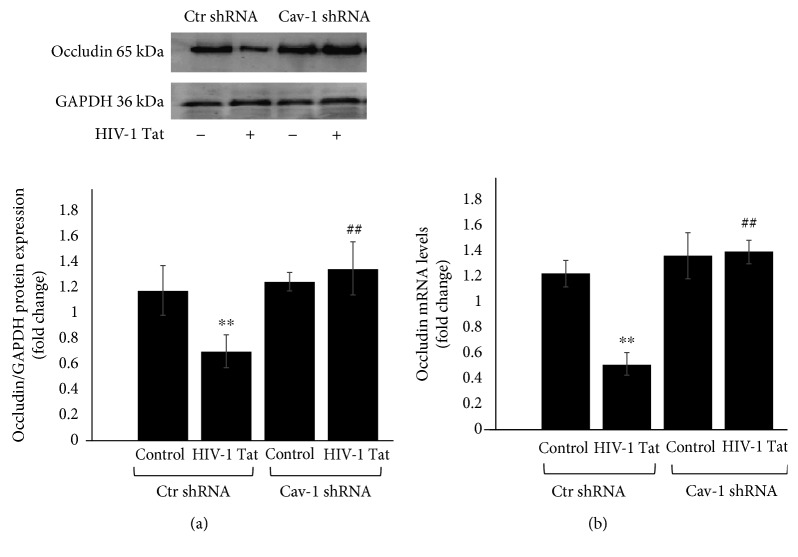
Role of Cav-1 shRNA in HIV-1 Tat-induced changes of occludin. Cav-1 expression in HBEC-5i cells silenced with Cav-1 shRNA or Ctr shRNA was exposed to HIV-1 Tat for 24 h. Occludin protein (a) and mRNA (b) levels were detected by Western blot or qRT-PCR, respectively. Occludin protein and mRNA levels in Ctr shRNA were decreased following HIV-1 Tat exposure but were upregulated in the Cav-1 shRNA group. Data are representative of three independent experiments. ^∗∗^*P* < 0.01 compared to Ctr shRNA. ^##^*P* < 0.01 compared to Ctr shRNA+HIV-1 Tat.

Cav-1 silencing by shRNA effectively protected against Tat-induced reduction in the expression of occludin [19]. To address the role of Cav-1 shRNA in HIV-1 Tat-induced changes of occludin, Cav-1 expression in HBEC-5i cells was silenced with Cav-1 shRNA or transduced with Ctr shRNA and exposed to HIV-1 Tat for 24 h. Occludin protein (Figure 3(a)) and mRNA (Figure 3(b)) levels were detected by Western blot and qRT-PCR, respectively. The occludin protein and mRNA levels in Ctr shRNA were decreased following HIV-1 Tat exposure (*P* < 0.01 in both Western blot and qRT-PCR) but were significantly upregulated in the Cav-1 shRNA group compared to Ctr shRNA+HIV-1 Tat (*P* < 0.01 in both Western blot and qRT-PCR).

### 3.4. The Role of Cav-1 shRNA in HIV-1 Tat-Induced Alterations of RAGE and LRP-1

**Figure 4:**
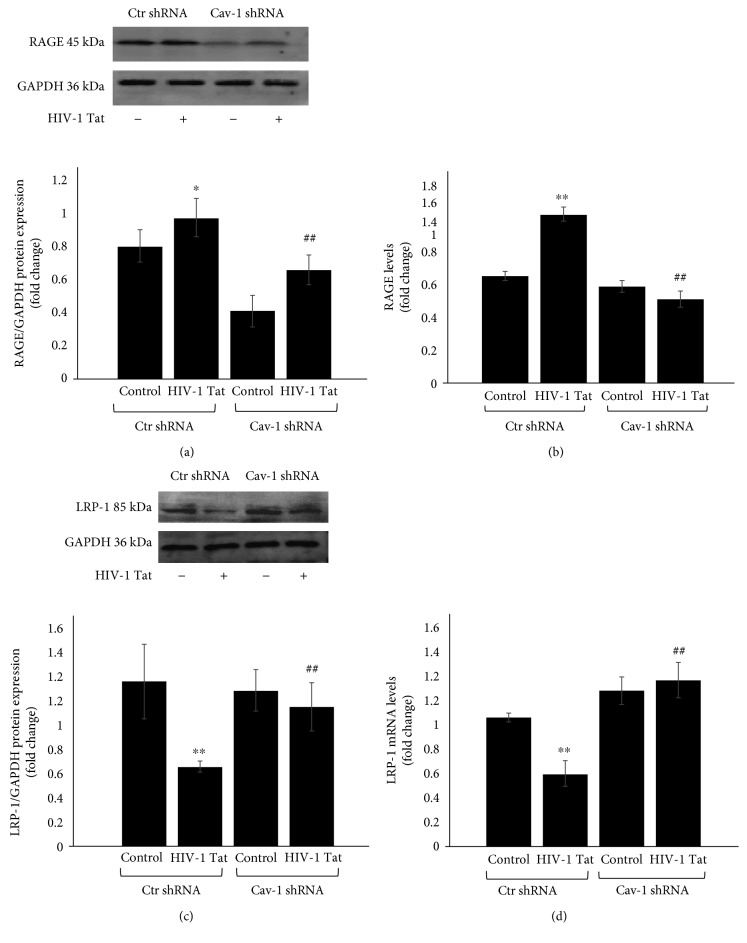
Role of Cav-1 shRNA in HIV-1 Tat-induced changes in RAGE and LRP-1. HBEC-5i with Cav-1 silencing by shRNA were exposed to HIV-1 Tat for 24 h. RAGE and LRP-1 protein and mRNA levels were detected by Western blot (a, c) and qRT-PCR (b, d). Compared with the Ctr shRNA, RAGE protein and mRNA levels were increased with HIV-1 Tat exposure but were downregulated in the Cav-1 shRNA group. Compared with the Ctr shRNA, LRP-1 protein and mRNA levels were downregulated by HIV-1 Tat exposure but were upregulated in the Cav-1 shRNA group. Data are representative of three independent experiments. ^∗^*P* < 0.05, ^∗∗^*P* < 0.01 compared to Ctr shRNA. ^##^*P* < 0.01 compared to Ctr shRNA+HIV-1 Tat.

In the HIV-infected brain, A*β* accumulates primarily as diffuse and intraneuronal deposits [22]. A*β* transport from the bloodstream into the brain is mediated by RAGE, while A*β* transport from the brain into the bloodstream is dependent on LRP-1. HBEC-5i cells in which Cav-1 expression was silenced with Cav-1 shRNA or transduced with Ctr shRNA were exposed to HIV-1 Tat for 24 h. RAGE and LRP-1 protein and mRNA levels were detected by Western blot (Figures 4(a) and 4(c)) and qRT-PCR (Figures 4(b) and 4(d)), respectively. Compared with the Ctr shRNA, the RAGE protein and mRNA levels were increased following HIV-1 Tat exposure (*P* < 0.05 in Western blot and *P* < 0.01 in qRT-PCR) but were downregulated in the Cav-1 shRNA group compared to Ctr shRNA+HIV-1 Tat (*P* < 0.01 in both Western blot and qRT-PCR). Compared with the Ctr shRNA group, the LRP-1 protein and mRNA levels were decreased following HIV-1 Tat exposure (*P* < 0.01 in both Western blot and qRT-PCR) but were upregulated in the Cav-1 shRNA group compared to Ctr shRNA+HIV-1 Tat (*P* < 0.01 in both Western blot and qRT-PCR).

### 3.5. The Role of Cav-1 shRNA in HIV-1 Tat-Induced Changes of RhoA

**Figure 5:**
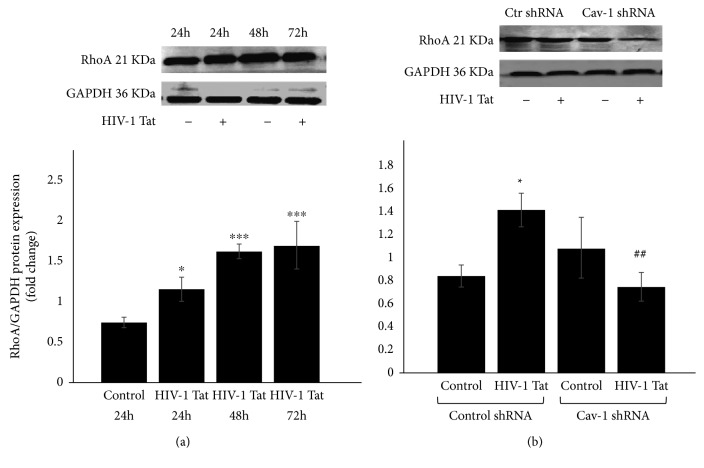
Role of Cav-1 shRNA in HIV-1 Tat-induced changes of RhoA. The RhoA protein levels were detected by Western blot. Compared with the blank control, HIV-1 Tat treatment increased the protein levels of RhoA with the longer exposure time (a). Compared with the Ctr shRNA, the RhoA protein level was upregulated following HIV-1 Tat exposure but was downregulated in the Cav-1 shRNA group (b). Data are representative of three independent experiments. ^∗^*P* < 0.05, ^∗∗^*P* < 0.01, and ^∗∗∗^*P* < 0.001 compared to Ctr shRNA. ^##^*P* < 0.01 compared to Ctr shRNA+HIV-1 Tat.

RhoA signaling has been linked to mural cell recruitment to the vessel wall in the brain, which is involved in the regulation of TJ protein integrity [23, 24]. Therefore, we also examined RhoA expression induced by HIV-1 Tat in the present study. The RhoA protein levels were detected by Western blot. Compared with the blank control, exposure to 1 *μ*g/mL of HIV-1 Tat resulted in a time-dependent increase in the protein levels of RhoA as illustrated in Figure 5(a). Compared with the Ctr shRNA, the RhoA protein level was upregulated following HIV-1 Tat exposure (*P* < 0.05) but was downregulated in the Cav-1 shRNA group compared to Ctr shRNA+HIV-1 Tat (*P* < 0.01, Figure 5(b)).

## 4. Discussion

Lipid rafts and caveolae play an important role in directing pathogen trafficking to specific sites. Disruption of lipid rafts by cholesterol depletion inhibits HIV entry into T cells. Caveolae play a regulatory role in the disruption of tight junction proteins in response to cellular exposure to HIV-1 Tat in HBMEC [19]. HIV-1 Tat disrupts neuronal homeostasis and may contribute to the neuropathogenesis in people living with HIV. Our previous studies have demonstrated that HIV-1 Tat may contribute to A*β* accumulation in brain endothelial cells by regulating the expression of the A*β* transfer receptors, RAGE and LRP-1 [16]. HIV-1 Tat also induces ZO-1 and neprilysin dysfunction in brain endothelial cells via the Ras signaling pathway [20]. However, the role of Cav-1 shRNA in HIV-1 Tat-induced dysfunction of tight junctions and the A*β*-transferring receptors RAGE and LRP-1 is not fully understood.

Cav-1, as the main structural protein of caveolae in endothelial cells, plays an important role in physiological and pathological processes of the BBB. The permeability of the BBB is connected with the participation of Cav-1. The expression of tight junction proteins is enriched in the caveola fraction of HBMEC, and Cav-1 silencing effectively protects against Tat-induced diminished expression of occludin [19]. Our findings support those of previous studies, as we show that the occludin protein and mRNA levels were upregulated in the Cav-1 shRNA group compared to the Ctr shRNA group following HIV-1 Tat exposure (Figure 3).

The BBB prevents the unregulated exchange of substances between the brain and blood. Peptides like A*β* generally do not cross the BBB, but they can be transported into the brain via the specific transferring receptors RAGE and LRP-1. The balance between RAGE and LRP regulates A*β* clearance through transport across the BBB. RAGE is the main A*β* influx transporter, transporting A*β* from the bloodstream into the brain, while LRP-1 is an A*β* efflux transporter, transporting A*β* from the brain into the bloodstream [25]. RAGE is upregulated in AD and is colocalized with A*β* in human AD brain tissues, neurons, microglia, and vascular elements [26, 27]. A disrupted BBB with decreased clearance of A*β* could be the underlying mechanism of brain A*β* accumulation. Exposure to HIV-1 elevates A*β* levels in brain endothelial cells, partly by increased expression of RAGE, which transports A*β* into the brain. However, the effects of HIV-1 on LRP-1 expression are inconsistent and, overall, did not reach statistical significance compared with the control [1]. It has been reported that LRP-1 regulates A*β* endocytosis in a caveolin-dependent manner [28]. In this study, the effect of Cav-1 silencing on HIV-1 Tat-induced RAGE and LRP-1 expression was investigated. Consistent with our previous data [16], exposure to HIV-1 Tat resulted in marked changes of RAGE and LRP-1 mRNA and protein levels. However, Cav-1 silencing with shRNA downregulated RAGE protein and mRNA levels, as well as upregulated LRP-1 protein and mRNA levels induced by HIV-1 Tat (Figure 4). Our data suggest that Cav-1 may be involved in preventing the transfer of A*β* into the brain and stimulate brain A*β* clearance across the BBB.

Extensive oxidative stress contributes to the progression of AD. A*β* may promote the formation of reactive oxidative species through the PI3K/Akt/GSK3 and MAPK/ERK1/2 pathways. LRP-1 also affects cellular A*β* uptake by a RhoA-dependent pathway [28]. The expression of the tight junction proteins occludin, ZO-1, and ZO-2 induced by HIV-1 Tat in caveolae is regulated via Ras signaling [19]. Cav-1 acts as a platform for signal transduction and cooperates with several signaling molecules by forming a complex [29]. As a Ras homolog gene family, RhoA is a small guanosine triphosphate-binding protein, and its activation plays a key role in modulating the cytoskeletal structure and permeability of endothelium [30]. The inhibition of p38 MAPK attenuates HIV- or Tat-mediated upregulation of Cav-1 [18]. Our previous study indicates the Rho inhibitor hydroxyfasudil attenuated HIV-1 Tat-induced decrease of occludin and LRP-1 expression and upregulation of RAGE in hCMEC/D3 cells [16]. The protein levels of RhoA increased in a time-dependent manner with HIV-1 Tat exposure in this study but were downregulated by Cav-1 silencing (Figure 5).

In summary, silencing the Cav-1 gene with shRNA significantly protects against HIV-1 Tat-induced downregulation of occludin and LRP-1 and upregulation of RAGE and RhoA, which may result in the accumulation of A*β* in the brain. Cav-1 plays a key role during HIV-1 Tat-induced dysfunction of the blood-brain barrier and A*β* accumulation. A better knowledge of the mechanisms involved in A*β* deposition in the brain and BBB dysfunction during HIV-1 infection will help to investigate new ways to reduce the A*β* burden in HAND.

## Data Availability

The Western blot and qRT-PCR data used to support the findings of this study are included within the article.
